# Ultracold cryogenic TEM with liquid helium and high stability

**DOI:** 10.1073/pnas.2509736122

**Published:** 2025-09-05

**Authors:** Emily Rennich, Suk Hyun Sung, Nishkarsh Agarwal, Maya Gates, Robert Kerns, Robert Hovden, Ismail El Baggari

**Affiliations:** ^a^The Rowland Institute at Harvard, Harvard University, Cambridge, MA 02138; ^b^Department of Mechanical Engineering, University of Michigan, Ann Arbor, MI 48109; ^c^Department of Materials Science and Engineering, University of Michigan, Ann Arbor, MI 48109; ^d^Michigan Center for Materials Characterization, University of Michigan, Ann Arbor, MI 48109; ^e^Department of Physics, University of Michigan, Ann Arbor, MI 48109

**Keywords:** liquid helium, cryo-TEM, electron microscopy, cryo-EM

## Abstract

Cryogenic transmission electron microscopy has revolutionized structural biology and materials science. To image below liquid nitrogen temperatures, various liquid helium stages have been constructed but have proven to be complex and unstable, making high-resolution imaging challenging. This problem is even more pronounced in side-entry specimen holders common on modern transmission electron microscopes. Here, we introduce an ultracold liquid helium transmission electron microscope side-entry specimen holder, featuring continuous cryogen flow and vibration decoupling. This instrument is compatible with modern aberration-corrected microscopes and achieves sub-25 K base temperature, ±2 mK thermal stability over many hours, and atomic resolution—setting the stage for a new era of cryogenic electron microscopy.

Rapid advancements of cryogenic transmission electron microscopy (TEM) techniques have revolutionized the biological (1, 2) and materials sciences (3–6) over the past decades. However, serious challenges persist in the realm of cryogenic TEM techniques, particularly when working at temperatures below 100 K. Ultracold temperatures below the boiling point of liquid nitrogen (77 K) in modern TEM are poised to improve the radiation tolerance and resolution in single-particle cryo-EM and cryoelectron tomography (7–11), and provide access to quantum phases in materials science research (12–16).

Dedicated helium stage modules were constructed and implemented early in the history of TEM (17–21); however, this approach is currently incompatible with high-resolution objective lens pole pieces due to the strict design constraints of modern electron optics. In life science applications, cartridge-based liquid nitrogen microscopes have become popular even though they lack ultracold performance and in situ capabilities. As an alternative, dewar-based side-entry cryogenic holders have filled the need to cool specimens to low temperatures. Unfortunately, the rapid evaporation of the cryogen within the dewar introduces large mechanical vibrations and thermal instabilities that result in poor imaging resolution. Moreover, the relatively small volume of these dewars leads to short operation times, or hold times (14, 15). While some of these challenges can be mitigated for liquid nitrogen, liquid helium has a heat of vaporization ∼60 times lower than that of liquid nitrogen, which dramatically increases instabilities and has made the use of liquid helium a persistent challenge for cryogenic TEM (15).

Here, we introduce a side-entry specimen holder capable of liquid helium cooling while achieving atomic resolution. This ultracold cryogenic TEM specimen holder demonstrates millikelvin temperature stability maintained for hours near liquid helium temperatures (<25 K at the specimen) in a modern aberration-corrected TEM. A base temperature of 23 K is maintained over 4 h. Moreover, the thermal stability is better than ±2 mK, a significant (estimated 10×) improvement over existing instruments. Thermal stability is directly associated with the reduction of specimen drift. We report mean drift speed of 0.37 Å/s; this is comparable to side-entry liquid nitrogen holders (0.75 Å/s), and approaches the stability of room-temperature holders (0.18 Å/s) (22). By maintaining stable cryogenic temperatures with liquid helium for extended durations, scientists can conduct intricate experiments without grappling with unpredictable temperature fluctuations or recurrent dewar refills.

Fig. 1*A* shows the atomic structure of gold at ∼31 K using this ultracold TEM specimen holder inserted into a double-corrected JEOL 3100R05 operating at 300 kV. This holder (Fig. 1*G*) employs liquid cryogen flow through a copper heat exchanger as the cooling source. Liquid helium continuously flows from a large external dewar along a vacuum-insulated and radiation-shielded transfer line to the copper heat exchanger. The sample is coupled to the cooled heat exchanger via highly thermally conductive components running axially inside the long hollow metal rod of the holder, keeping it cold as long as liquid helium is flowing. The extended surface area of these cold components act as internal cold traps in the vacuum space, preventing ice accumulation on the specimen even after multiple hours of experiments. No noticeable ice buildup was observed during the over 24 h experiments in Figs. 1 and 2 using JEOL 3100R05 at $∼7.5\;\times \;10^{-8}$ torr column pressure.

**Fig. 1.**
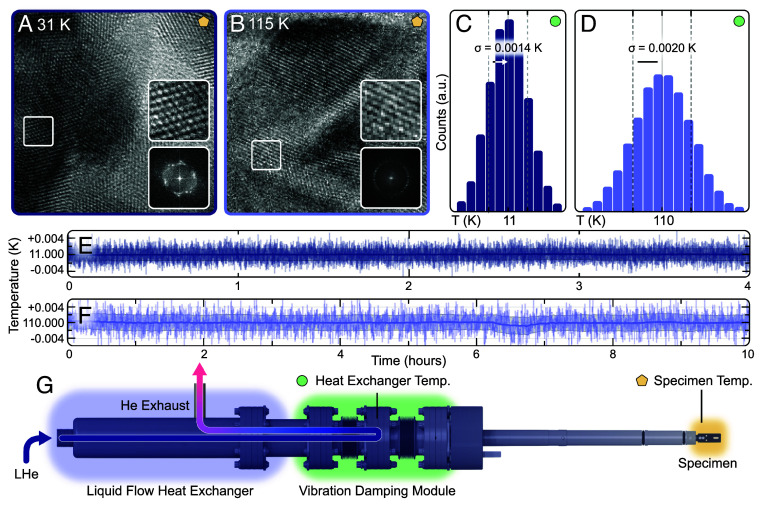
Ultracold cryogenic TEM imaging at atomic resolution with mK temperature stability. (*A* and *B*) Atomic resolution image of gold at 31 K, and 115 K, respectively. The *Top Insets* represent a zoom-in into the TEM images. *Bottom Insets* show the Fourier transform of the atomically resolved TEM images. (The scale bar represents 2 nm.) (*C* and *D*) Histograms of measured sensor temperatures at 11 K and 110 K, respectively. A temperature stability of ±1.4 mK was measured at a heat exchanger base temperature of 11 K over a data collection period of 4 h. A temperature stability of ±2.0 mK was measured at an intermediate heat exchanger temperature of 110 K over a data collection period of 4 h. (*E* and *F*) Plot of the measured heat exchanger temperature fluctuations as a function of time at 11 K and 100 K, respectively. Multihour long hold times with ≤2 mK stability were demonstrated at both temperatures. (*G*) Rendered model of the instrument components including the cryogen-flow heat exchanger, the vibration damping module, and TEM specimen tip. Liquid helium flow through the heat exchanger is denoted using an arrow with relative holder temperature denoted by a blue-white gradient overlay. Temperature measurements were taken using a calibrated silicon diode sensor at the specimen tip and liquid flow heat exchanger. Temperature difference between heat exchanger and specimen tip is ∼15 K.

**Fig. 2.**
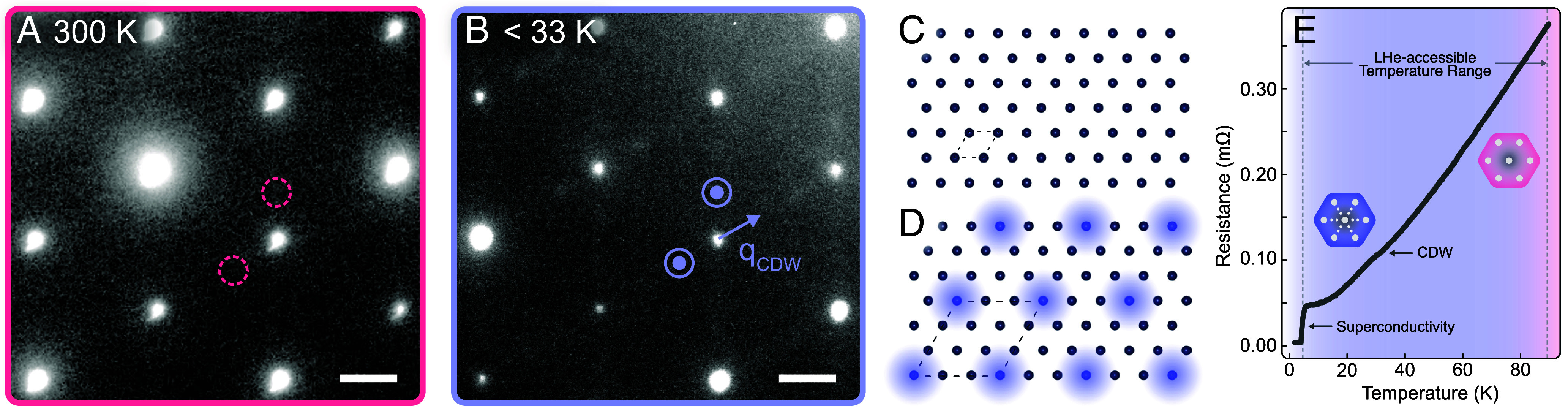
(*A*) TEM electron diffraction pattern captured at 300 K shows the room temperature phase containing only Bragg peaks corresponding to a normal hexagonal crystal lattice. (*B*) At temperatures below 33 K superlattice peaks (blue circles) appear, indicating emergence of charge density waves with a threefold supercell. The 1/3 reciprocal lattice unit wavevector is represented by a blue arrow. (*C*) Simplified model of the hexagonal atomic arrangement of Nb atoms (dark circles) in 2H-NbSe_2_. This corresponds to the diffraction pattern in (*A*). Dashed lines represent the unit cell at high temperature. The lattice constant representing the Nb-Nb distance is 0.34 nm. (*D*) Model of the atomic arrangement overlaid of the charge density wave superlattice (dashed lines). The light blue circles represent the extra charge density that forms a supercell at low temperature. This corresponds to the diffraction pattern in (*B*). (*E*) Temperature-dependent resistance curve shows the emergence of electronic phase transitions including a CDW transition at ∼33 K and superconductivity below 7 K. The CDW transition appears as a slight anomaly in the temperature–resistance curve whereas the superconducting transition appears as a sudden drop to zero resistance. (The scale bars represent 2 nm^−1^.)

Vibrations are mitigated by decoupling the specimen from the helium transfer line through two pairs of flexible ultrahigh vacuum edge-welded bellows with a spring constant of 4.7 N/mm. Each bellow compresses 50A durometer rubber blocks that dampen vibrations emanating from the transfer line and room environment. The flexible bellows also accommodate movements of the microscope’s goniometer, enabling the usual positional adjustments in the x, y, and z axes, as well as axial rotation.

In addition to low base temperatures, this liquid helium cryogenic TEM holder allows precise control over a wide range of temperatures without compromising thermal stability. By adjusting the flow of helium and applying carefully controlled heating, the holder can be stabilized anywhere between room and base temperature with a stability of ±2 mK or better. This capability has been demonstrated, reaching ±1.4 mK and ±2.0 mK stability at temperatures of 11 K and 110 K, respectively (Fig. 1*C*–*F*). This instrument is also cryogen-agnostic: Liquid nitrogen can be used instead of liquid helium if only temperatures above 110 K are needed.

Millikelvin stability over 10 h is shown in the temperature plot (Fig. 1*F*) at an intermediate temperature of 110 K, though similarly long hold times are possible at any operating temperature. In fact, hold times are only limited by the available helium supply—this holder consumes helium at a rate of less than two liters per hour. Commonly available helium tanks are between 30 to 1,000 L, making long hold times easily achievable. These long hold times present a substantial opportunity for measurements that require long experimental durations including single particle reconstruction, chemical spectroscopy, tomography, and ptychography.

The ability to easily reach intermediate temperatures also affords a great deal of flexibility: materials can now be reliably characterized at any desired temperature, not just near the boiling point of liquid nitrogen. We note that this instrument does not currently implement shielded cryotransfer. However, being a side-entry holder, cryotransfer methods could be implemented in the future using approaches similar to existing side-entry liquid nitrogen holders. This would expand the range of examinable specimens and enable single particle reconstructions of vitrified biological molecules.

Beyond applications for the preservation of beam-sensitive specimens, ultracold TEM opens up avenues for investigating quantum materials, crystalline solids in which novel electronic states emerge at low temperatures. An illustrative example is the observation of a structural phase transition below 33 K in the layered quantum material 2H-NbSe_2_ (Fig. 2), which further validates ultracold TEM performance. 2H-NbSe_2_ is a layered transition-metal dichalcogenide that exhibits charge density wave (CDW) ordering below 33 K and superconductivity below 7 K (23). CDW formation is characterized by the appearance of superlattice peaks in reciprocal space. Fig. 2*A* shows an electron diffraction pattern at 300 K from a thin exfoliated flake. Bragg peaks with the hexagonal symmetry of the crystal are prominent. Accessing temperatures below 33 K with the ultracold holder, we observe the emergence of additional superlattice peaks (Fig. 2*B*, circles) indicating the formation of the CDW. These peaks are located at 1/3 reciprocal lattice units, the predicted wavevector for CDW in 2H-NbSe_2_.

The results herein represent a significant advancement that addresses a longstanding desire to access ultracold temperatures within a modern aberration-corrected electron microscope, offering opportunities for probing challenging biological and quantum materials. Cryogenic electron microscopy at ultracold temperatures may prove beneficial to improving radiation tolerance and imaging resolution of key materials underlying biological and renewable energy research (24). This development also enables a field for characterization of quantum materials, including mapping of phase transitions such as superconductivity at temperatures unattainable with liquid nitrogen cooling.

## Supplementary Material

Appendix 01 (PDF)

## Data Availability

All study data are included in the article and/or *SI Appendix*.
