# Supplementary material for: Ultracold cryogenic TEM with liquid helium and high stability
Source: Proc Natl Acad Sci U S A. 2025 Sep 5;122(36):e2509736122. doi: 10.1073/pnas.2509736122 (PMC12435197; doi:10.1073/pnas.2509736122)
Supplement: Supplementary file 1 — Appendix 01 (PDF) [file pnas.2509736122.sapp.pdf]

Supporting Information for

Ultra-Cold Cryogenic TEM with Liquid Helium and High Stability

Emily Rennich, Suk Hyun Sung, Nishkarsh Agarwal, Maya Gates, Robert Kerns, Robert Hovden, and Ismail El Baggari

Ismail El Baggari
E-mail: ielbaggari@fas.harvard.edu

This PDF file includes:

Supporting text

Supporting Information Text

Methods

Cryogenic Experiment. The holder incorporates liquid helium flow cooling and flexible thermal and vacuum connections to reduce mechanical vibration coupling. The internal cryogenic components operate under high vacuum conditions provided by the microscope column vacuum ($\sim 7.5\times10^{-8}$ torr). Due to the increase in internal surface area, the holder is pumped under vacuum for at least 30 minutes before imaging. Internally, a copper heat exchanger is connected to a copper braid and rod assembly that traverses the bore of the TEM holder. Because the alignment of the overall assembly is horizontal, mechanical supports for the internal copper components are necessary. In this case, the supports consist of alternating layers of vacuum-compatible polyetheretherketone (PEEK) nets and Mylar sheets. These materials not only exhibit low thermal conductivity but their layered configuration creates a winding thermal pathway and limited thermal contact to the warm outer jacket of the TEM holder.

A liquid helium transfer line connects the specimen holder to a large external helium source—here a 60 L stainless steel dewar was used. The dewar is depressurized when the helium transfer line is inserted and secured to the dewar. Pressure in the dewar is then raised and maintained at $\sim$5 psi to ensure flow into the ultra-cold specimen holder.

For precise temperature control, we use a flexible heating element attached to the heat exchanger to maintain the desired temperature. The temperature is measured using a silicon diode for general cryogenic use from 1.4 K to 500 K with an excitation DC current of 10 μA and controlled using a temperature controller (Cryo-Con Model 22C) with a proportional-integral-derivative (PID) scheme. A second silicon diode temperature sensor is mounted at the tip, near specimen to monitor specimen temperature.

Transmission Electron Microscopy. The in situ cooldown was performed on a double Cs-Corrected JEOL3100R05 STEM/TEM equipped with tungsten cold-FEG operated at 300 kV held under a $\sim 5.2\times10^{-11}$ torr vacuum. The microscope column pressure of $\sim 7.5\times10^{-8}$ torr was maintained around the specimen throughout the experiment. TEM images (Fig. 1a,b) were acquired on Gatan Ultrascan1000 CCD camera with 25 ms acquisition time. Electron diffraction patterns were collected using an 850 nm selected area aperture.

Specimen Preparation. Specimens were mounted to a copper tip using indium solder to maximize thermal transport. Sputtered gold on an ultrathin carbon film covered a standard 3 mm copper mesh grid. 2H-$NbSe_2$ was exfoliated onto polydimethylsiloxane (PDMS) gel stamp and mechanically transferred using a home-built transfer system onto silicon-based TEM grids. The silicon based TEM grids contain a SiN_x membrane window TEM grid with 2 μm holes and were produced by Norcada.

Temperature-Dependent Resistance Measurements. Temperature-dependent resistance measurements on 2H-$NbSe_2$ were performed in a 1.8 K cryostat with a superconducting magnet (Quantum Design PPMS DynaCool). Samples were prepared by exposing a fresh surface of 2H-$NbSe_2$ by scotch-tape exfoliation and thin gold wires were attached using silver paint. Resistance measurements were done in a 4-probe configuration with a 1 mA current being sourced from a Keithley 6220 Current Source and voltage measured via a Keithley 2812A Nanovoltmeter. The sample was cooled in helium exchange gas. The measurements shown here were performed under a 2 T out-of-plane magnetic field to match the magnetic field experienced by the sample in a TEM. Due to this magnetic field, the superconducting transition is suppressed to $\sim$5 K instead of the $\sim$7 K transition temperature under ambient conditions. The charge density wave transition is unaffected by the magnetic field.
